# Supplementary figures and images for: Role of the SAF-A/HNRNPU SAP domain in X chromosome inactivation, nuclear dynamics, transcription, splicing, and cell proliferation
Source: PLoS Genet. 2025 Jun 10;21(6):e1011719. doi: 10.1371/journal.pgen.1011719 (PMC12176297; doi:10.1371/journal.pgen.1011719)

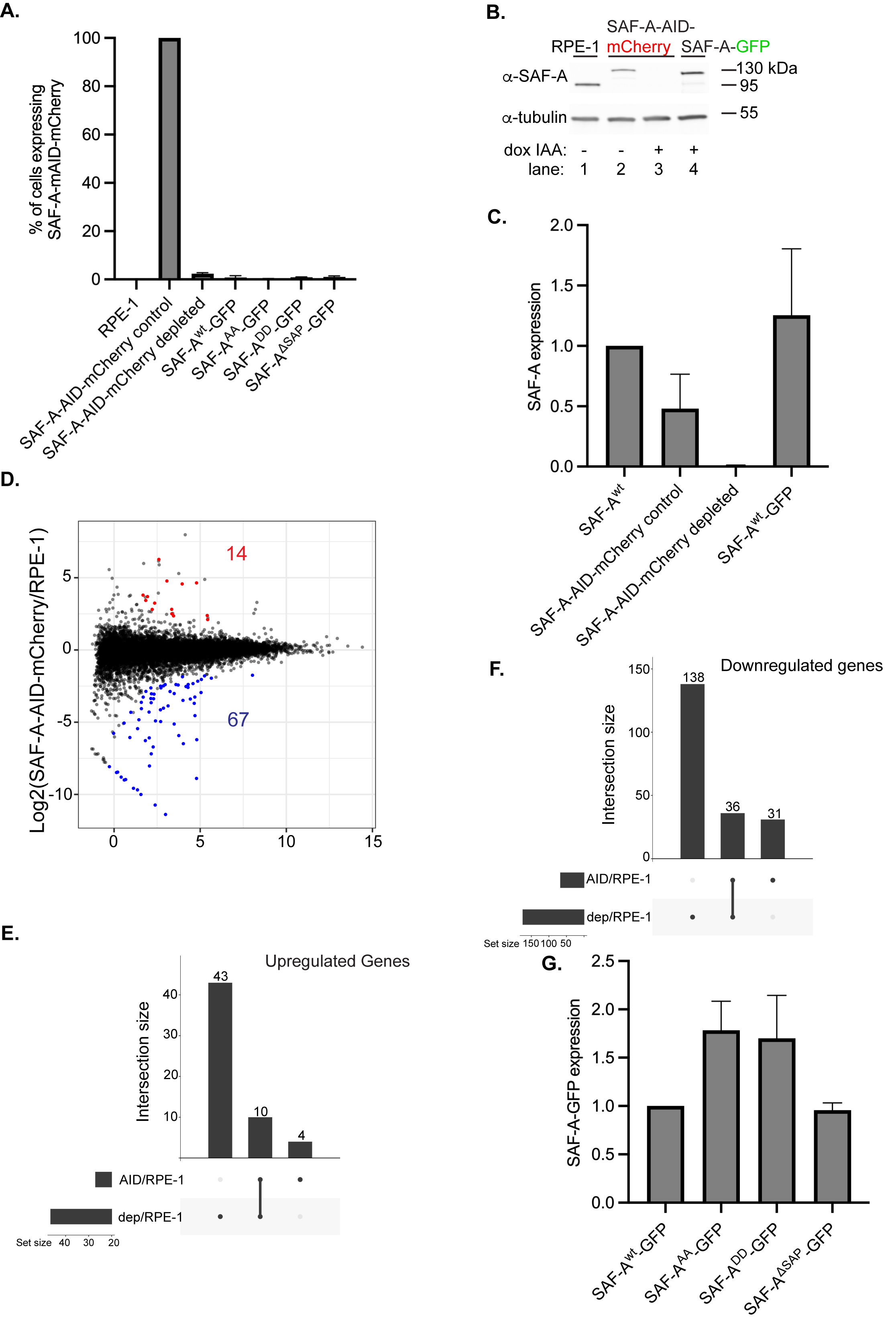

Supplement: S1 Fig — A. Cells were scored for SAF-A-AID-mCherry expression after 24 hours of drug treatment. The graph depicts the average percent of cells (n = 100) expressing the degron allele; error bars depict the SD for two biological replicates. B. Western blot analysis with the mouse anti-SAF-A 3G6 monoclonal antibody to compare levels of endogenous SAF-A (lane 1), SAF-A-AID-mCherry -/ + drug treatment (lanes 2 and 3) and SAF-Awt-GFP (lane 4). C. Quantitation of SAF-A expression levels relative to the tubulin loading control in three different extract preparations. Error bars depict SD. D. MD plot comparing gene expression between wt RPE-1 cells and RPE-1 cells with the SAF-A-AID-mCherry expressed from the endogenous locus. Differentially expressed genes are highlighted in red and blue. E-F. Upset plots comparing the intersection of misregulated genes in untreated SAF-A-AID-mCherry cells to cells depleted of SAF-A by the addition of dox and IAA. G. Quantitation of SAF-A-GFP expression levels. We were unable to identify an antibody that recognizes all tagged versions of SAF-A and SAP domain mutations. Instead, we monitored expression levels of GFP-tagged SAP domain mutations using a GFP antibody and compared to the SAF-Awt-GFP control to infer approximate expression levels relative to the endogenous protein. We estimate expression levels of all GFP tagged transgenes is within a 2-fold difference compared to the endogenous protein. (TIF) [file pgen.1011719.s001.tif]

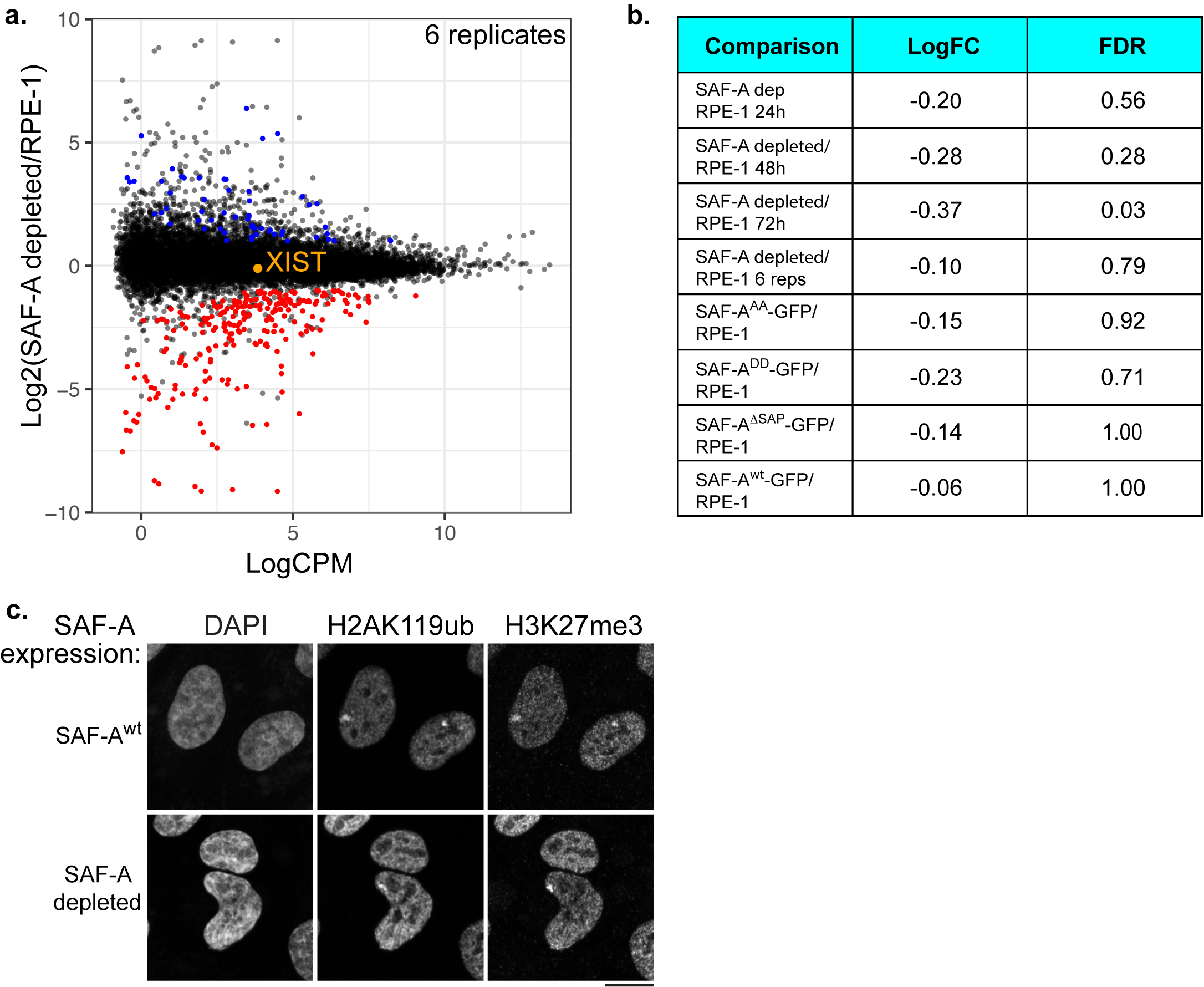

Supplement: S2 Fig — A. MD plot comparing gene expression in 6 replicates of SAF-A depleted cells to wild type RPE-1 cells. XIST RNA expression is highlighted in orange. B. Table depicting the logFC and FDR of XIST RNA for all SAF-A depletion and mutant experiments as measured by RNA-seq. C. Immunofluorescence of H3K27me3 and H2AK119ub in SAF-Awt and SAF-A depleted cells. Scale bar is 10 µm. (TIF) [file pgen.1011719.s002.tif]

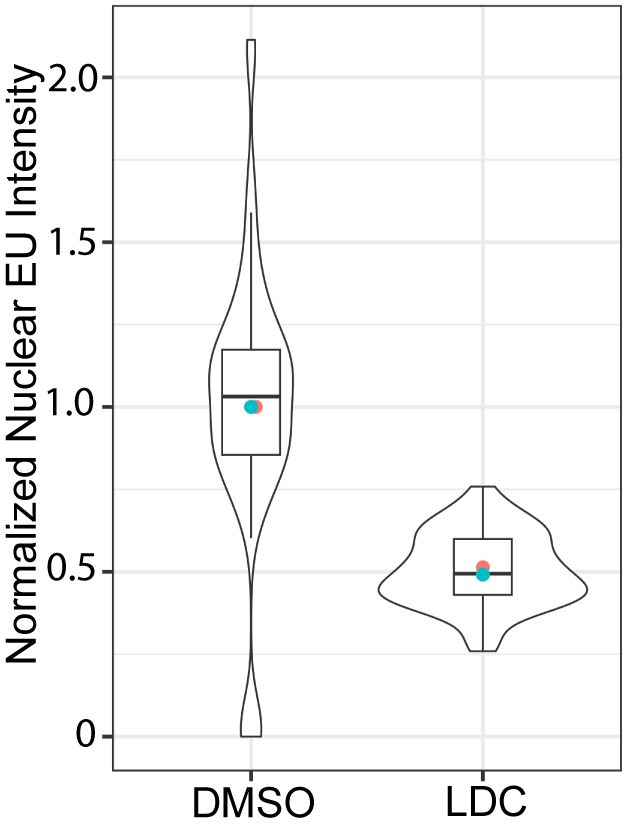

Supplement: S3 Fig — Cells were incubated with the CDK9 inhibitor LDC, or DMSO, for 30 minutes prior to the addition of EU. Nuclear EU fluorescence intensity was measured from two biological replicates. (TIF) [file pgen.1011719.s003.tif]

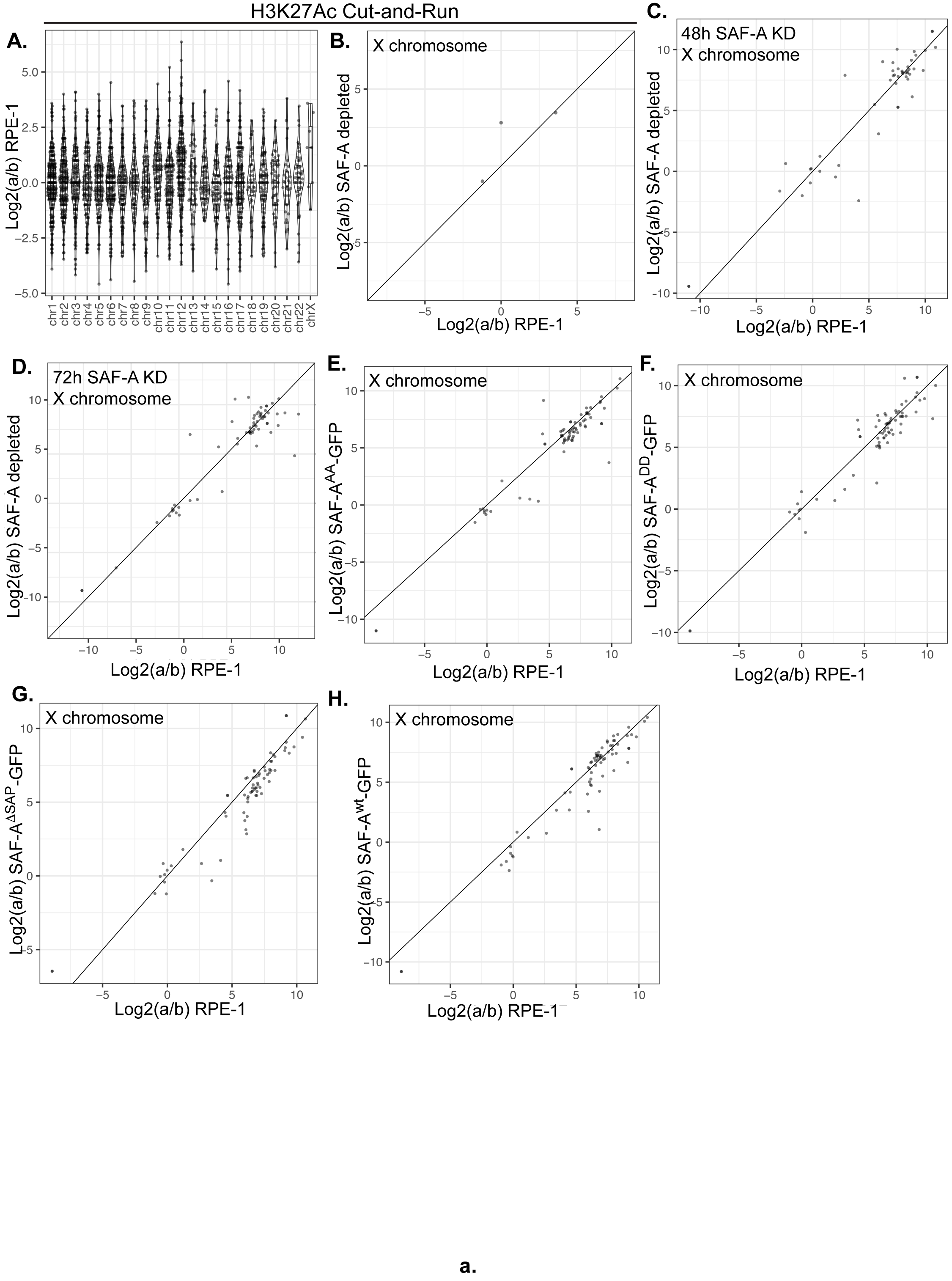

Supplement: S4 Fig — A. Allele-specific Cut-and-Run was performed with H3K27Ac antibodies and calculated using PAC. ‘a/b’ ratios are plotted for each gene by chromosome. B. Comparison of ‘a/b’ ratios for X linked genes in SAF-A depleted cells. C. Comparison of ‘a/b’ ratios for all X-linked genes after 48 hours of SAF-A depleted cells. D. Comparison of ‘a/b’ ratios for all X-linked genes after 72 hours of SAF-A depletion. E-H. Comparison of ‘a/b’ ratios for all X-linked genes after in all SAF-A SAP domain mutants. (TIF) [file pgen.1011719.s004.tif]

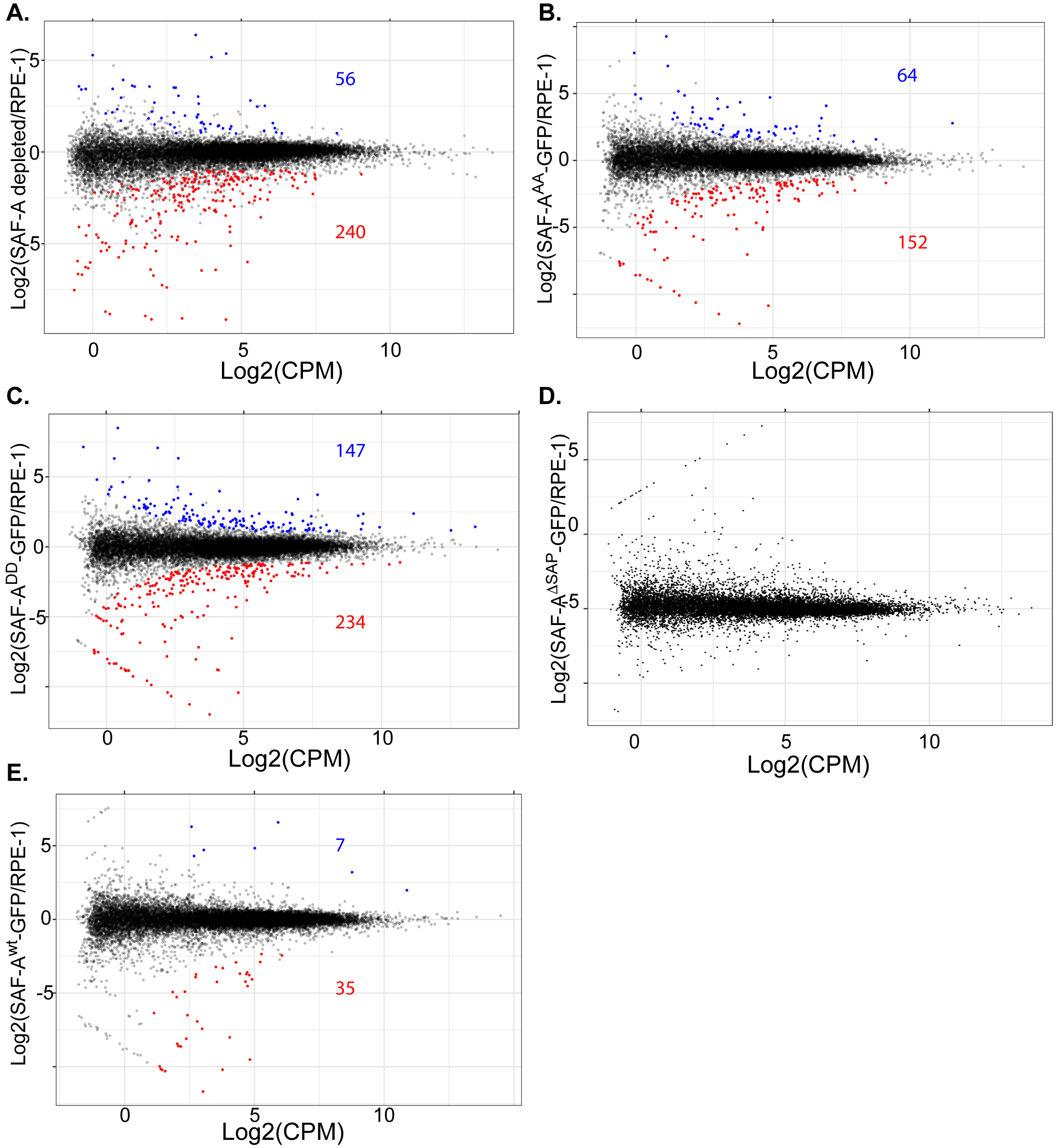

Supplement: S5 Fig — A-E. Gene expression was evaluated at 24 hours after addition of doxycycline and auxin, using RNA-seq and EdgeR. MD plots depict significantly differentially expressed genes for each mutant (FDR < 0.01). The gene expression profile of cells depleted for SAF-A, or expressing SAF-A alleles SAF-Awt-GFP, SAF-AAA-GFP, SAF-AAA-GFP, or SAF-AΔSAP-GFP were compared to RPE-1 cells as indicated on the y axis. (TIF) [file pgen.1011719.s005.tif]

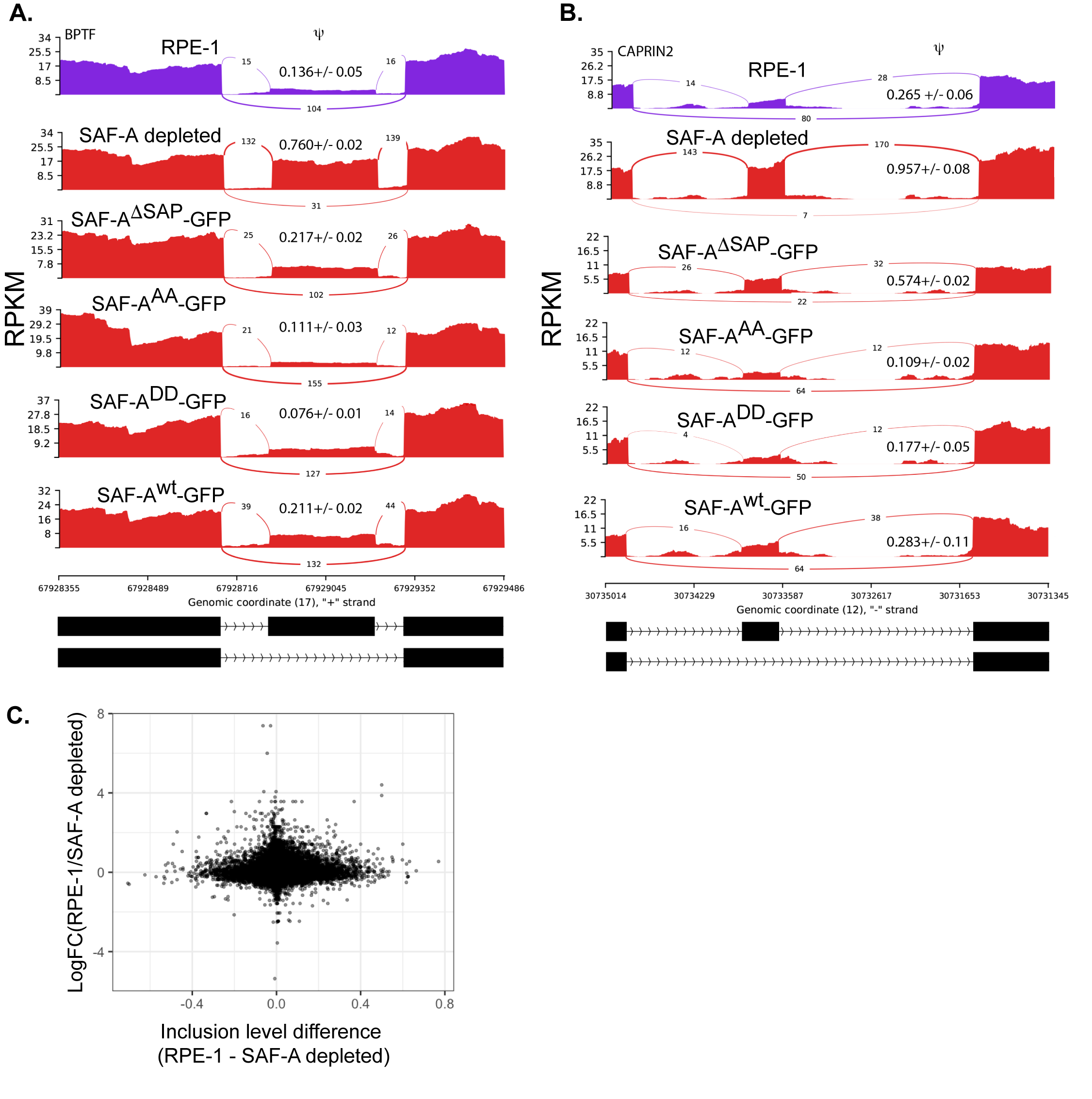

Supplement: S6 Fig — A-B. MISO plots of SE events in WT RPE-1 cells, SAF-A depleted cells, and SAP domain mutants. C. Scatterplot comparison of changes in gene expression at 24h to changes in mRNA splicing. (TIF) [file pgen.1011719.s006.tif]

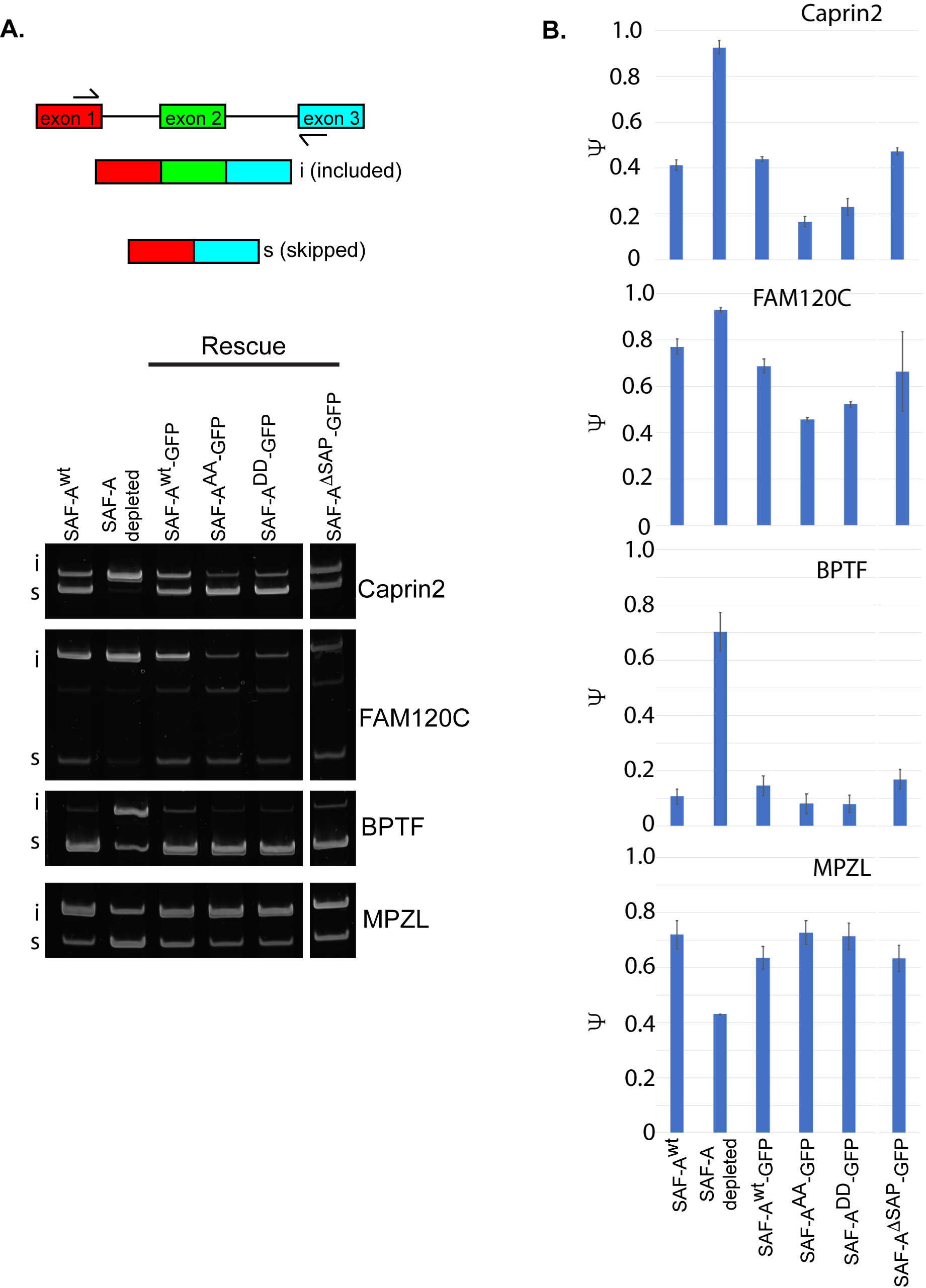

Supplement: S7 Fig — A. Illustration depicting included and skipped exons, and PAGE gel analysis of exon inclusion for 4 different genes with predicted changes in exon inclusion in SAF-A depleted cells. B. Quantitation of percentage spliced in in each mutant from three biological replicates. (TIF) [file pgen.1011719.s007.tif]

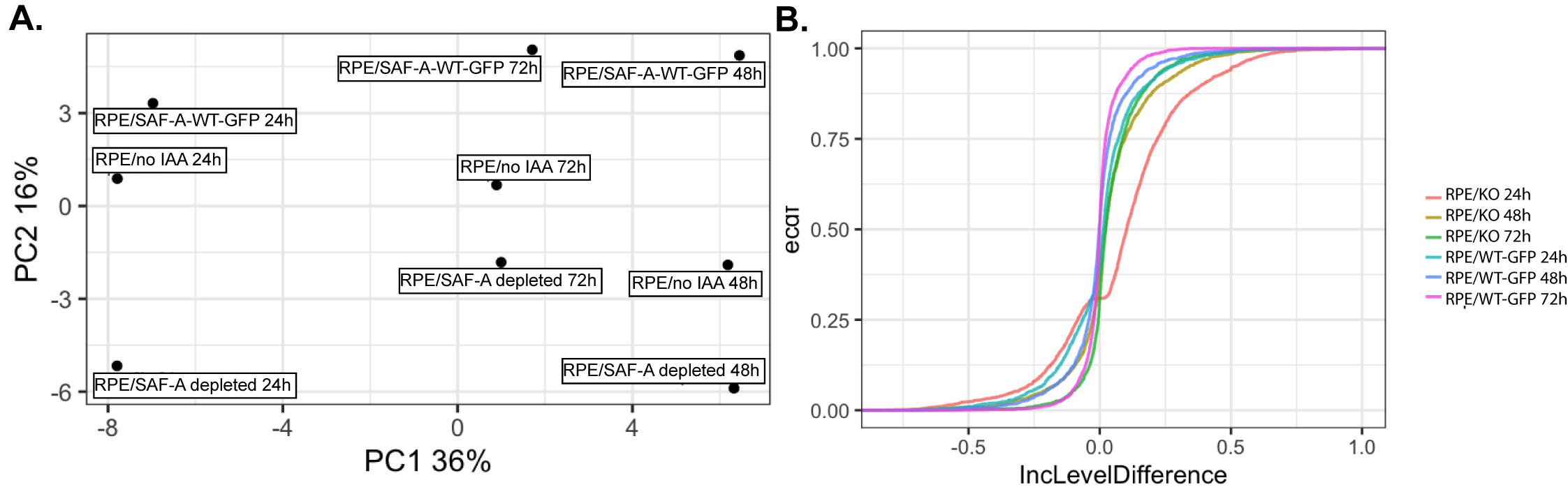

Supplement: S8 Fig — A. PCA analysis of skipped exons (SE) in SAF-A depleted cells, RPE-1, and SAF-A depleted cells rescued with SAF-Awt-GFP at each time point. B. CDF analysis of SE altered in SAF-A depleted cells at 24 hours in each cell type at each time point. (TIF) [file pgen.1011719.s008.tif]

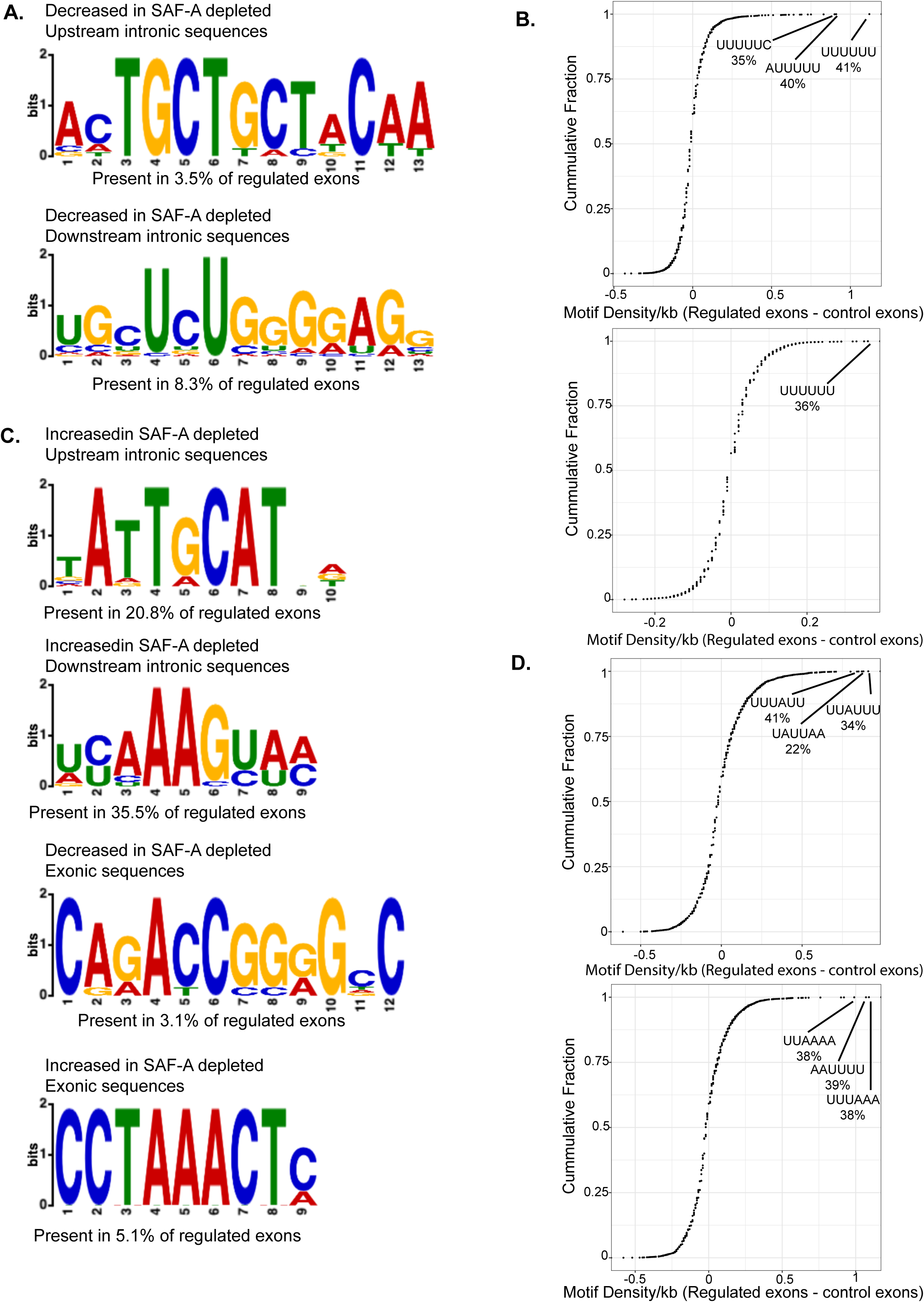

Supplement: S9 Fig — A. STREME analysis of upstream and downstream intronic sequences surrounding SAF-A regulated exons. B. Hexamer enrichment in the same sequences as in A. C-D. STREME and hexamer analysis of sequences in and surrounding SAF-A regulated exons. Sequence sets tested are indicated on the plots. (TIF) [file pgen.1011719.s009.tif]

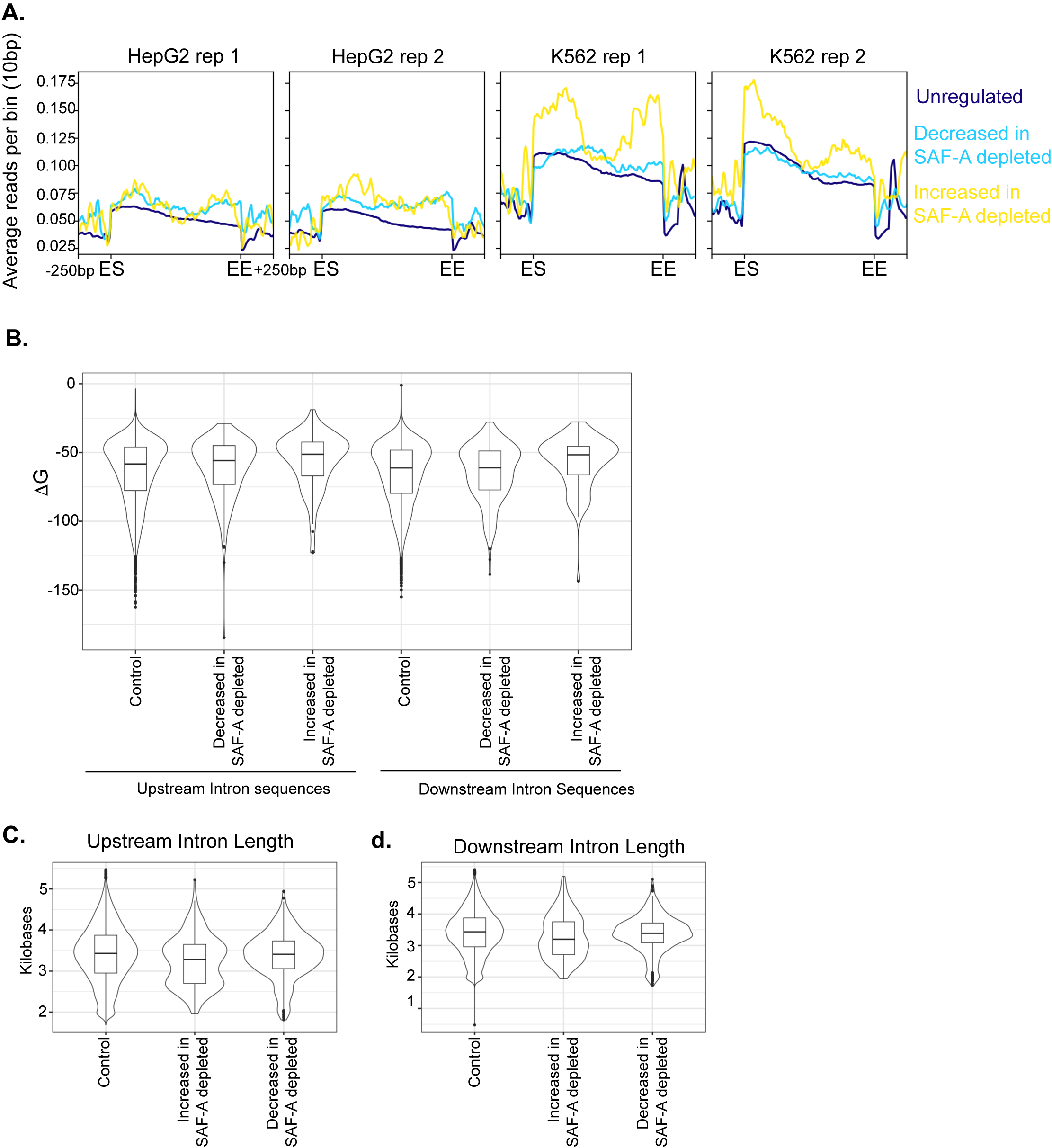

Supplement: S10 Fig — A. ENCODE project eCLIP data was analyzed using Deeptools in both HepG2 and K562 cells at SAF-A regulated exons (increased or decreased) and at unregulated genes. Average coverage and heatmap are depicted for all datasets. B. mFold calculation of ΔG for indicated sequenes. C-D. Calculated lengths of indicated sequence groups. (TIF) [file pgen.1011719.s010.tif]
