# Supplementary material for: Role of the SAF-A/HNRNPU SAP domain in X chromosome inactivation, nuclear dynamics, transcription, splicing, and cell proliferation
Source: PLoS Genet. 2025 Jun 10;21(6):e1011719. doi: 10.1371/journal.pgen.1011719 (PMC12176297; doi:10.1371/journal.pgen.1011719)
Supplement: Attachment — Submitted filename: Reponse to Reviewers 3-30-25.pdf [file pgen.1011719.s022.pdf]

Response to reviewers:

We thank the reviewers for their critical and constructive reading of our manuscript. We are pleased that all reviewers considered our analysis of the SAF-A/HNRNPU SAP domain to be a potentially useful contribution to the literature. All three reviewers raised concerns with the initial version of our manuscript. In the revised manuscript we have addressed all the concerns raised by each reviewer with new experiments, additional data analysis, and substantial rewriting of the manuscript. Outlined below are our responses to each point raised by the reviewers.

Comments to the Authors:
Please note here if the review is uploaded as an attachment.

Reviewer #1: Overall this is an interesting manuscript which highlights the role of the SAP domain of HNRNPU in XIST RNA localisation and RNA splicing. The experiments are in general well controlled and executed. There are some areas where better representation of the existing literature would allow the reader to see a better context for the new data.

1. HNRNPU is the official gene name and the name used in databases so it makes the article very inaccessible to persist in using the terminology SAF-A. To broaden access of this article it is important to include HNRNPU in both the title and abstract, otherwise many searches from scientists who came across this protein through a database would miss your article.

We have made the changes. (p.1, line 1 and p.2 line 13)

2. Fig. 1d . It would be helpful to understand the extent of constitutive degradation caused by addition of the mini-Aid to the HNRNPU ORF by probing the Western blots with anti-HNRNPU antibody as well so we can see to what extent the – auxin condition is reflective of WT cells and to what extent the GFP fusions used for complementation are overexpressing HNRNPU relative to normal endogenous levels. Whilst I recognise it is challenging to mimic endogenous levels, overexpression may itself cause phenotypes (see for example Table 1 in PMID: 10490622). So, it would be helpful for the reader to understand any limitations of the system being used. Whilst the authors state they monitored transgene levels after induction with tet, it would be nice to see the actual data. Especially when they make this statement in the discussion “. The stable integration of inducible SAF-A transgenes allowed for precise titration of SAF-A expression level, in order to approximate normal protein levels as closely as possible.” I note also that in the original paper describing the generation of the RPE HNRNPU-AID cell line again no Western was provided showing HNRNPU levels probed with a HNRNPU antibody compared to the native RPE cells, only +/- auxin conditions of the engineered cell line. Finally I note for the RNAseqs the authors used RPE vs HNRNPU depletion as their two conditions, rather than -/+ auxin. Was this because the -auxin condition already had substantial depletion of HNRNPU due to the well-known constitutive degradation caused with some proteins tagged with auxin degrons?

We have tested 4 different antibodies to endogenous SAF-A to measure relative expression of the wild-type protein, SAF-A-mAID-mCherry, and SAF-A-GFP transgenes. We have not been able to identify a single antibody that recognizes all tagged and mutant isoforms. For example, rabbit a-SAF-A (abcam 20666) recognizes endogenous SAF-A but not the SAF-A-AID-mCherry tagged version. The 3G6 mouse a-SAF-A antibody (Santa Cruz sc-32315) recognizes endogenous SAF-A, SAF-A-AID-mCherry, SAF-A wt-GFP, and SAF-A-AA-GFP, but not SAF-A-DD-GFP or SAF-A-DSAP-GFP. Two additional polyclonal antibodies (Bethyl Laboratories) were tested without success.

Because the 3G6 antibody can detect SAF-A-mAID-mCherry and SAF-A-GFP, we used this antibody to compare expression levels of the wild-type and Cherry/GFP-tagged isoforms. SAF-A-mAID-mCherry constitutive expression was approximately half that of wild-type; addition of doxycycline and auxin resulted in complete depletion of the degron allele. SAF-A-wt-GFP expression was an average of 1.2-fold above the wild-type protein, quite close to normal physiological levels. (Supplemental Figure S1b-c).

Because of the challenge in identifying a single antibody that recognizes all tagged and mutant isoforms, instead we used a GFP antibody to quantitate relative expression of SAF-A-GFP and GFP-tagged SAP domain mutations (Supplemental Figure 1b-c and g.) From this analysis we can infer that the integrated SAF-A-GFP transgenes are all expressed at near physiological levels, within a 2-fold difference relative to the endogenous protein. We have included the blot quantitation in the text (p. 5 lines 87-91, 97-98) and changed the sentence in the discussion (p. 14, lines 337-338).

We appreciate Reviewer 1's question asking why we chose the RPE-1 parental line for genetic comparisons. The SAF-A-mAID-mCherry allele can be cultured in perpetuity and shows similar growth to wild-type cells, suggesting it complements for essential functions of SAF-A. However, the analysis with the 3G6 antibody suggests that steady-state levels of SAF-A-mAID-mCherry may be lower than the endogenous protein, perhaps due to intrinsic stability differences. To more fully answer Reviewer 1's question, we performed gene expression analysis of SAF-A-mAID-mCherry cells (-dox -IAA) and compared to RPE-1 wild-type cells, and found a small number of differentially expressed genes in the SAF-A-mAID-mCherry cell line (Supplemental Figure 1e). Based on these two observations, the parental RPE-1 wt cell line offers the best baseline wild-type condition available for comparison to our GFP-tagged rescue lines.

3. Fig. 1h . Are the DD, AA and delta SAP mutants in fact acting in a dominant negative manner since these cells seem to perform worse in both assays than the HNRNPU depleted condition?

At present we do not know why there is a difference in penetrance of the cell cycle phenotype for SAP domain alleles compared to the null condition. Theoretically it could be possible that the SAP mutations are acting in a dominant-negative manner, possibly by changing binding properties or sequestering associated factors. Without a known mechanism we are unsure whether to call the alleles dominant in this assay, since that would imply a gain of function, and the cell cycle exit phenotype is observed in the SAF-A depleted cells (a loss of function mutation). It therefore might be more appropriate to label the SAP mutations LOF/recessive mutations, with different degrees of penetrance observed at each time point. We have included a statement in the revised manuscript to help clarify this issue (p.5 lines 106-108).

4. In discussing the data presented in figures 3b-d it would probably be worth mentioning that HNRNPU was defined as a HNRNP protein, all of which bind nascent, intronic RNA, moreover, a key RNA binding domain of HNRNPU is the C-terminal RGG box, in fact the phrase was first termed on the basis of its discovery in HNRNPU (PMID:1628625). Therefore, the observation that “there is a population of SAF-A molecules that are stably associated with nascent transcripts independently of the SAP domain (Figure 3d)” is consistent with the prior literature, including the prior work of these authors.

This is a helpful point and we have made the change (p. 15 lines 367-368).

5. Fig. 5. RNA-seq does not measure transcription. It measures the steady state levels of the transcriptome which can be influenced by multiple factors including transcription and decay rates. The text should reflect this and not infer effects on transcription.

We have made the change (p. 11 line 265 and 284).

6. Fig S4 what do I and S mean? Perhaps a cartoon showing where the primers are on the gene structure and what is being amplified would be helpful for the reader.

Thank you for pointing this out. The letters mean: I = mRNA isoform with included exon S = mRNA isoform with skipped exon. We have added a cartoon and informative titles to the figure and explanations to the figure legend. (Figure S6a.)

7. The discussion comments about HNRNPU being transiently associated with nuclear elements including RNA should be discussed in the context of its well known role as a HNRNP protein, which bind heterogeneous nuclear RNA i.e nascent RNA, which is, in general, chromatin associated via the transcribing polymerase it is exuding from. Furthermore, it has previously been shown that HNRNPU depletion leads to increased levels of U2 snRNP in the cell PMID: 22325991 and that this could account for the major changes in splicing seen following HNRNPU depletion. Especially given the binding sites for HNRNPU on pre-mRNA correlate poorly with splicing changes, implying a general effect on the splicing machinery is at play as suggested in PMID: 22325991. Such a conclusion is consistent with the data presented here in Fig. S7 and should be considered in the discussion.

We have changed the text to explicitly mention the U2 snRNP mechanism (p. 11 line 288; p. 13 lines 328-330).

Minor points:
1. Should LDC be described as a CDK9 inhibitor rather than a polII inhibitor

We have changed the text to reflect that LDC is a CDK9 inhibitor (p. 8 lines 192).

2. p.14 line 23- Refers to Figure 5e, there is no figure 5e.

We have removed this error from the text.

P12 line 23- "normal" RPE-1 cells feels like a vague description, maybe qualify with "diploid" or "karyotypically normal" something to that effect. They use "normal, diploid" in the discussion

We modified to wording to describe RPE-1 cells as ‘diploid, karyotypically stable’ instead of normal (p. 5 line 82, p. 14 line 333).

3. p.17 line 26- close bracket "(Figure 3 and [20],"

Fixed

4. Supplemental figures:
-inconsistent bolding of first "A." left over from bolding title in 4 and 7. Fixed
Supplemental figure 1 legend- "C.F" should be "C-F" to match formatting of other legends Fixed
Supplemental figure 5- More descriptive/legible names for the figure legend would help readability, at least some underscores or dashes

Thank you for pointing this out. We have added informative labels to the plots.
Supplemental figure 6 legend- replace "C. C-D" with "C-D" Fixed

5. Methods- Can't find a description of how they've lysed their cells for the knock down validation western blot in results or methods.

We have included this information in the revised methods (p. 19 lines 463-477).

6. References
p.28:There seems to be multiple places where fragments of the competing interests are inserted into references, see 3. 11. and 13. p.31

We removed these fragments from the references.

p.8:
line 8- Reference 17 doesn't seem relevant to the statement (unless I'm mistaken).

We removed this reference.

Reviewer #2:

In the manuscript ‘Nuclear compartments have distinct requirements for the SAF-A SAP domain’ the authors aim to characterise the effect of the N-terminal SAP domain on the functionality of SAF-A by generating a homozygous SAF-A-mCherry auxin mediated degron cell line in human RPE cells, and then complementing these cells with stable heterozygous integration of either WT SAF-A-GFP or a SAF-A mutant-GFP to the TRE3Gs locus. Expression of either a SAP domain deletion, a SAP AA mutant or a SAP DD phosphomimetic mutant is induced with the use of doxycycline.

The authors present limited novel findings in this manuscript: They recapitulate the previous finding that depletion of SAF-A has an effect upon splicing, but none of the SAP domain mutants demonstrate this phenotype. Furthermore, transcriptomic analysis shows minimal effects of SAF-A depletion or expression of mutants unless cells are maintained for 48 or 72 hrs in the absence of SAF-A. As the authors demonstrate in Figure 1 that by this time such cells already exhibit significant cellular stress with 20-40% of cells in G0, and a clear decrease in replication, any analysis of transcription is likely to be unreliable. Likewise, any comparison of genes affected by splicing with genes that show aberrant transcription after 48 or 72hrs is unreliable for the same reason.

SAF-A has several functional domains and is involved in several essential cellular functions. Our approach in this paper was to focus on one domain and characterize its contribution to multiple processes. While our work shows that the SAP domain is not the primary determinant of the role of SAF-A in splicing, our system allows for identifying introns that are directly and immediately impacted by acute depletion of SAF-A, which is a novel contribution.

Another novel finding in our paper is the unexpected result that most of the cellular pool of SAF-A shows rapid nuclear movement, engaging with nuclear structures in a dynamic manner. This is a major correction in the field, since previous studies proposed SAF-A to be part of a rigid structure based on crude nuclear fractionation experiments. We note that Nick Gilbert and

colleagues have come to similar conclusions and have SAF-A FRAP data in their preprint manuscript (https://www.biorxiv.org/content/10.1101/2024.06.16.599208v2.full.pdf).

We also identify a novel role for SAP domain phosphorylation contributing to chromatin structure on the inactive X chromosome, and to prevent cell cycle exit.

Finally, we appreciate the reviewer drawing attention to the transcription data, and interpreting that there are cell cycle changes contributing to gene expression at longer time points. This speaks to a major point that we have attempted to make with this manuscript, that SAF-A depletion has acute effects on splicing, XIST localization, and maintenance of Polycomb histone modifications, while large-scale effects on gene expression are only observed after long-term depletion of SAF-A. We conclude that SAF-A does not play a direct role in regulating gene expression and have included this interpretation in the Discussion. We have speculated about the cause of the cumulative gene expression changes in the results (p. 11 lines 276-278) and in the discussion: "It is possible that this occurs through an indirect mechanism, such as a misexpressed gene or misspliced RNA causing long term amplification of gene expression changes. It is also possible that SAF-A affects a low turnover element of chromatin, such as a long-lived histone modification or chromatin modifier." (p. 15-16 lines 388-390).

A previous publication demonstrated that the mouse SAP domain was required for Xist localisation. A further publication identified Ser14 and 26 in the SAP domain of SAF-A as important during mitosis. The novel data in this manuscript relates to the recruitment of SAF-A and Xist to the inactive X in interphase human cells and its reliance upon dynamic phosphorylation of S14 and S26. This information is a useful contribution to the field, but there are major points which need to be addressed:

Further major points:

1) The authors suggest that there is ‘complete knockdown of endogenous SAF-A in the majority of cells after 24hrs’. Western blot analysis showing the full degree of knockdown after 24hrs should be shown, particularly as a proportion of cells can be seen to express SAF-A mCherry even after 24hr IAA treatment in Figure 1e.

This is of further importance in Figure 1h, where complementation with the panel of mutant transgenes has more effect upon %PCNA positive cells and %Ki-67 positive cells than depletion of SAF-A in its entirety. This might be explained if there were a dominant negative effect on residual endogenous SAF-A mCherry.

The Western blot analysis requested by the reviewer was depicted in Figure 1d, in the a-RFP panel. The antibody recognizes the mCherry protein since it is a derivative of RFP. We have relabeled the blot to make this clear. In our revised manuscript we have added quantitative western blot analysis using the 3G6 monoclonal antibody (Supplemental Figure 1b-c). Both these analyses demonstrate the quantitative depletion of endogenous SAF-A protein. For more detailed information please also see our response to Reviewer 1 point #2.

The image shown for SAF-A-AID-mCherry depleted cells in Figure 1e shows a small number of cells in the population that continue to express the protein after drug treatment, which is not unusual for AID cell lines. We quantitated the percent of cells that show mCherry expression in

SAF-A-AID-mCherry depleted cells and SAF-A-GFP rescue lines and found that >98% of cells had depleted the degron allele. We have included this quantitation as new Supplemental Figure 1a.

Taken together, we have documented mCherry expression in our cell lines by imaging fields of cells and providing quantitative scoring. We have measured mCherry expression in cell extracts using two different antibodies. All data support the claim that there is "complete knockdown of endogenous SAF-A in the majority of cells" after 24 hours of drug treatment.

Since we have not found evidence for residual levels of SAF-A-AID-mCherry we do not think this would be responsible for the variable penetrance of the cell cycle phenotype among SAP mutants compared to SAF-A depleted cells. Please also see our response to Reviewer 1 point 3.

2) The authors state multiple times that transgene expression is equivalent to endogenous expression levels, but this is not shown ('unpublished data' in methods). This is an important omission from both the western blot and IF data.

We have performed quantitative Western blots comparing GFP-rescue transgenes to endogenous levels of SAF-A in RPE-1 cells and in SAF-A-AID-mCherry cells. This new data in included in a new Supplementary Figure 1b-d, g.
We have addressed this point using a combination of antibodies that recognize endogenous SAF-A/HNRNPU and a GFP antibody to recognize the rescue transgenes. For the details of our findings please see response to Reviewer 1 point #2.

3) In figure 2 the authors use analysis of 3D confocal stacks of Xist foci. They suggest that as they are unable to resolve Xist RNA particles normally localised to the Xi with confocal microscopy, an increase in particles distal to the Xi would correlate to more particles that they can resolve. This approach is flawed as it does not distinguish between increased Xist molecules per nucleus versus the same number of Xist molecules that are more dispersed.

We have analyzed XIST expression in all of our RNA-seq experiments (2 replicates each at 24, 48, and 72 hours from the time course depletion of SAF-A; 6 replicates at 24 hours from various SAF-A depletion experiments, and 2 replicates each of depletion and rescue with each mutant reported in the manuscript) and detect no changes in XIST expression. This data is included in a new Supplemental Figure 2. We conclude that changes in XIST localization are not a result of increased expression.

Furthermore, in Figure 2a, delocalised Xist can be seen in examples provided, but if the images are representative they must be highly thresholded, for example in SAF-A depleted cells, the

average Xist foci in the graph is 12 (range approx. 4-30), but approx. 35 foci are evident by eye! Likewise, the idea that an average of 4 Xist RNA foci are evident in the SAF-A WT cells is clearly so inaccurate that the axis should be re-labelled Xist aggregations.

The images provided in Figure 2a are not thresholded. The method that we used to detect XIST particles is not capable of resolving individual particles that are very close because we are using conventional confocal microscopy, not super-resolution microscopy. As a result, very close XIST particles are merged into a single object during the particle identification step. Importantly we have used the identical particle detection algorithm on all images and have compared mutants. We are not claiming to to be capable of counting individual XIST molecules, but showing that the relative particle number changes following SAF-A depletion and in many mutants. We have altered the wording of this section to be clear that we are not presenting numbers of XIST molecules, but only of particles detected by our algorithm (p. 6, lines 125-126).

We also wish to emphasize that our aim was to identify a quantitative method to score XIST localization among several genotypes without the significant cost of using a super-resolution microscope. Our analysis (Figure 2b) demonstrates that our method detects reproducible, statistically significant differences between control samples and the SAF-A depletion, therefore establishing the validity of this approach.

4) The authors do not present data addressing whether SAF-A GFP mutants are equally capable of binding chromatin as endogenous SAF-A. Even the FRAP data in Figure 3c/d does not address this as the dynamics of the GFP transgenes is not compared to that of endogenous SAF-A with an mCherry tag.

Without further analysis of general chromatin binding, they cannot make definite claims that recruitment to the Xi is specifically affected.

We agree with Reviewer 2 that the FRAP data is not unambiguously reporting SAF-A interactions with chromatin. To address Reviewer 2's question of whether SAF-A SAP domain mutants have different chromatin binding properties, we tried two different approaches. First, we attempted to identify DNA/chromatin binding sites throughout the genome using the CUT&Tag assay. In all experimental replicates we were able to prepare high quality libraries and sequence them. However, we were able to call very few (<50) 'peaks' of SAF-A enrichment, suggesting that SAF-A does not exhibit stable binding to any sites in the genome. We performed this assay with two different antibodies and multiple replicates, but observed very little peak overlap between replicates. Unfortunately, because these results cannot distinguish between a failed CUT&Tag assay and a failure of SAF-A to bind stably to specific DNA sequences in the genome, we have not included this data in the revised manuscript.

Next, we assayed for SAF-A interaction with general chromatin by testing for the presence of histone H3 in SAF-A-GFP immunoprecipitation eluates (new Figure 3e-f). The data suggest that

the SAP domain mutants do not have differential chromatin binding properties in cells during interphase. Based on this data, it is unlikely that chromatin/nucleosomes on the Xi are involved in recruitment to the Xi compartment. We have tried to clarify this point in the text (p. 9 lines 245-251). Instead we speculate SAP domain regulation could be impacting protein-protein interactions or protein-RNA interactions.

5) The authors suggest there is a 'Xi-targeting signal or sequence in the SAP domain, that is largely independent of Ser 14 and 26', and 'distinct from other nuclear territories', but provide no evidence for such sequence. Targeting might be mediated by protein structure and / or interaction with an accessory protein and no other functionality that requires chromatin binding is addressed.

The imaging data shown in Figure 3a shows that a SAF-A SAP domain deletion causes exclusion from the Xi compartment in all cells analyzed. We did not observe this in cells expressing mutations at positions S14 and S26 (AA and DD mutants). Therefore, our genetic analysis of cis elements required for SAF-A Xi localization does support the conclusion that other sequences among the remaining 32 amino acids in the SAP domain are responsible for the Xi localization phenotype. Our imaging showed no other areas of the nucleus that displayed unique exclusion of the SAF-A-ΔSAP-GFP mutation, supporting the statement that the Xi compartment is distinct from other nuclear territories. We agree that our genetic analysis is focused on cis elements rather than trans interactions with accessory proteins. As detailed above, we have added language to the manuscript to clarify the SAP domain could be impacting protein-protein interactions or protein-RNA interactions.

6) The authors cannot argue that 'dynamic phosphorylation is required for the formation of a functional, mature Xist RNP' as they present no evidence to suggest that Xist RNP formation is aberrant in any fashion, only that there is a defect in its recruitment / retention.

We have changed the wording of this section to indicate that this is speculation (p.14 lines 353-354).

Minor points:

1) Details of the macro used for analysis of Xist particles should be provided to enable readers to reproduce experiments

We have included the ImageJ Macro and Perl program used to count XIST particles.

2) Figure 2c. Please include the SAF-A WT control and SAF-A depleted images that correlate to data the graph in Figure 2d.

These images are now included in Supplemental Figure 3.

3) Figure 2c/d. Please correlate uH2A / K27 enrichment with an Xi marker – otherwise this data is based on educated guesswork as to where the Xi might be.

It is established in the literature that H3K27Me3 and H2AK119Ub are highly concentrated in the Xi in human RPE-1 cells (PMID 33745031, 38181737, 12082075, 15574503, 30103804, 23542155, 25057298), which is consistent with a large number of studies from mouse cells. Additionally, we have provided images here demonstrating that the nuclear concentration of H2AK119Ub colocalizes with the Xi marker macroH2A in wild-type RPE-1 cells Reviewer Figure 1, below). Further, all Xi markers we have tested are disorganized in SAF-A depleted cells and SAF-A SAP domain mutants. These include XIST RNA, H3K27me3, H2AK119ub, and three other protein markers for the Xi. Therefore the assay in Figure 2c-d is scoring presence or absence of a large chromosome-sized enrichment of polycomb marks in cell populations. The images shown are projections, so each optical slice of the whole cell is present in the image, to illustrate that the Xi polycomb mark enrichment is absent throughout the entire nuclear volume in approximately half of the cells.

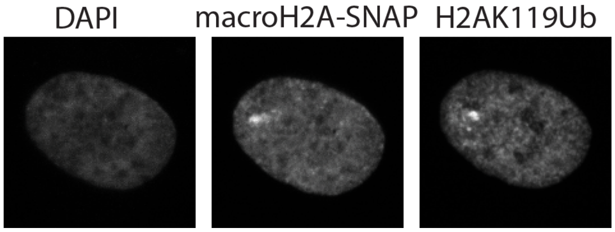

7) The accompanying text to Figure 3a suggests there is enrichment of the SAF-A DD mutant on the Xi, but to a lesser extent to the SAF-A AA mutant. There is not enrichment evident in the example. Furthermore, it would be useful for the proportion of cells in which SAF-A Xi enrichment / exclusion is observed to be detailed.

We have clarified the language for the sentence in question. (p. 8 line 173) We have added the proportion of cells to the figure legend.

8) Figure 5. Please explain further why interruption of SAP phosphorylation would have more of an effect than deletion of the whole SAP domain on gene expression?

The SAF-A S14 S26A phosphomutant and S14D S26D phosphomimetic allele have a complex genetic profile, exhibiting recessive/loss of function phenotypes in some assays and dominant/gain of function phentotypes in others (e.g. during mitosis). It is possible that the modest transcription changes observed in the phosphomutant and phosphomimetic alleles may

occur due to different binding properties or sequestration of protein binding partners, causing nonproductive complexes. At present though we do not know the molecular mechanism for the genetic observation.

9) When RNA-seq data from the RPE cells is analysed based on haplotype analysis in Figure 4 how many genes are able to be analysed on the X compared to the autosomes with this SNP based approach – it would appear far fewer on the X chromosome versus autosomes.

We have measured the SNP density of the X chromosome and found that it is ~1/2 the density of the other chromosomes. The number of genes that we can accurately measure allele-specific expression varies depending on the experimental technique and depth of sequencing reads. For RNA-seq with good sequencing depth (~200 million reads) we can accurately measure allele specific expression of ~70 genes on the X chromosome. A lower density of SNPs on the X chromosome is also observed in the haplotype-resolved genome of IMR90 cells (PMID 36869353), which have approximately ½ the SNP density on the X chromosome compared to autosomes.

10) The title is too generalised. The authors have not to my mind demonstrated that ‘nuclear compartments have distinct requirements for the SAF-A SAP domain’.

We have changed the title to specifically describe the content of the paper. (p.1 lines 1-2).

Reviewer #3:

This study focuses on the SAF-A protein’s SAP domain and its phosphorylation in nuclear processes. Using a degron-based system in human RPE-1 cells, the authors show that SAF-A is highly dynamic, transiently interacting with chromatin, RNA, and other nuclear structures. They identify phosphorylatable serines (S14, S26) in the SAP domain as critical for maintaining XIST RNA localization on the inactive X chromosome, enabling PRC1/PRC2-dependent histone modifications, and ensuring normal cell proliferation. Although depleting SAF-A affects mRNA splicing, the SAP domain itself appears to have limited influence on this process. Overall, these findings suggest that SAP domain phosphorylation drives rapid SAF-A turnover in distinct nuclear compartments, providing new insights into regulatory layers of nuclear organization and function. The manuscript is generally well written and concise, with data that largely support the authors’ conclusions. However, the lack of page, line, and figure numbers complicates the review process.

The results will interest researchers in heterochromatin and RNA biology, even though the SAP domain’s physiological impact appears modest in RPE-1 cells. With a few specific issues addressed, this manuscript should be suitable for publication in PLOS Genetics.

Major Points:
1. The authors claim complete endogenous SAF-A depletion with auxin and doxycycline but

only show Western blots using anti-fluorescent protein antibodies, not antibodies against endogenous SAF-A. Additionally, there is no genotyping data confirming both alleles are tagged with no remaining untagged allele. To meet PLOS Genetics standards, the authors should provide an antibody that recognizes endogenous SAF-A (if available) and present molecular validation of the knock-in strategy.

In our previous publication (Sharp et al. JCB 2020) we used PCR to validate the homozygous knock-in to the SAF-A/HNRNPU locus in RPE-1 cells. We have now provided Western blots using an antibody against endogenous SAF-A to show that the knock-in is homozygous and that auxin addition results in complete SAF-A degradation. The new data is provided in Supplemental Figure 1a-c.

2. In Figure 2, the conclusion that Xi targeting is defective is based on a more dispersed XIST signal. The authors should rule out the possibility that this dispersion is due to increased XIST levels rather than mislocalization.

We have used RNA-sequencing to show that SAF-A depletion or mutation do not result in increased XIST RNA levels. This new data is provided in Supplemental Figure 2.

3. The authors use LDC to block transcription but do not include controls confirming effective transcriptional inhibition. Such a control should be provided.

We have measured the efficiency of LDC transcriptional inhibition by measuring EU incorporation. Consistent with our previous work (36790906) and the work of others we find that a short incubation results in a strong decrease in EU incorporation into nascent transcripts. This new data is provided as Supplemental Figure 3.

4. The authors mention that gene expression changes accumulate over time, but they do not show whether the same sets of genes are affected at different timepoints. Demonstrating overlap in misregulated genes would strengthen their conclusions.

Thank you for this suggestion. We have calculated the intersection of misregulated genes at 24, 48, and 72 hours. We find that most of the misregulated genes at 24 hours are also misregulated at 48 and 72 hours. Later time points accumulate new misregulated genes. This new data is now included in a modified Figure 5.

5. The authors frequently cite figure panels out of sequence (e.g., referring to panel c before panel b). Citing panels in order would improve readability and clarity.

We have updated the Figure panel ordering and text callouts to refer to panels in order whenever possible. There are a couple of exceptions, but it is much improved from the original version.

Minor Points:

• In Fig. 2d, the authors should provide statistical analyses.

We have performed statistical analysis and included p-values in the Figure 2 legend (p. 24 lines 619-627).

• In the FRAP experiments, why authors do not examine SAF-A dynamics specifically at the Xi? It could provide direct evidence of distinct turnover in this compartment.

We have attempted this experiment but have been unsuccessful so far. To achieve the high frame rate needed for FRAP there is a tradeoff in resolution such that the Barr body is hard to identify. We would need a setup where we can identify the Xi without bleaching the sample before FRAP. We likely need to use a spinning disc microscope equipped with a separate bleaching laser, but currently do not have access to an appropriate microscope.

In Fig. S2, the authors should include the number of misregulated genes, as done in Fig. 5.

We have added these numbers to the plots. The figure is renumbered in the revised manuscript as Supplemental Figure 5.

• The terminology for SAF-A degration (e.g., “depletion” vs. “knockdown”) should be used consistently throughout the manuscript to avoid confusion.

We have removed "knockdown" from the text to avoid confusion. Thank you for pointing this out.
